# Clinical predictors for bradycardia and supraventricular tachycardia necessitating therapy in patients with unexplained syncope monitored by insertable cardiac monitor

**DOI:** 10.1002/clc.23594

**Published:** 2021-03-16

**Authors:** Tatsuya Onuki, Makoto Shoji, Hiroto Sugiyama, Shuhei Arai, Kosuke Yoshikawa, Hiroshi Mase, Masaaki Kurata, Miwa Kikuchi, Daisuke Wakatsuki, Taku Asano, Hiroshi Suzuki, Kaoru Tanno, Youichi Kobayashi, Toshiro Shinke

**Affiliations:** ^1^ Division of Cardiology, Department of Medicine Showa University Tokyo Japan; ^2^ Division of Cardiology, Department of Medicine Showa University Fujigaoka Hospital Yokohama Japan; ^3^ Cardiovascular Center Showa University Koto Toyosu Hospital Tokyo Japan

**Keywords:** insertable cardiac monitor, predictor, unexplained syncope

## Abstract

**Background:**

Insertable cardiac monitors (ICMs) improve diagnostic yield in patients with unexplained syncope. The most of cardiac syncope is arrhythmic causes include paroxysmal bradycardia and supraventricular tachycardia (SVT) in patients with unexplained syncope receiving ICM. Predictors for bradycardia and SVT that necessitate therapy in patients with unexplained syncope are not well known.

**Hypothesis:**

This study aimed to investigate predictors of bradycardia and SVT necessitating therapy in patients with unexplained syncope receiving ICMs.

**Methods:**

We retrospectively reviewed medical records of consecutive patients who received ICMs to monitor unexplained syncope. We performed Cox's stepwise logistic regression analysis to identify significant independent predictors for bradycardia and SVT.

**Results:**

One hundred thirty‐two patients received ICMs to monitor unexplained syncope. During the 17‐month follow‐up period, 19 patients (14%) needed pacemaker therapy for bradycardia; 8 patients (6%) received catheter ablation for SVT. The total estimated diagnostic rates were 34% and 48% at 1 and 2 years, respectively. Stepwise logistic regression analysis indicated that syncope during effort (odds ratio [OR] = 3.41; 95% confidence interval [CI], 1.21 to 9.6; *p* = .02) was an independent predictor for bradycardia. Palpitation before syncope (OR = 9.46; 95% CI, 1.78 to 50.10; *p* = .008) and history of atrial fibrillation (OR = 10.1; 95% CI, 1.96 to 52.45; *p* = .006) were identified as significant independent predictors for SVT.

**Conclusion:**

Syncope during effort, and palpitations or history of atrial fibrillation were independent predictors for bradycardia and for SVT. ICMs are useful devices for diagnosing unexplained syncope.

## 1 INTRODUCTION

Syncope has various causes, and the prognosis differs according to the cause.^1^ When conventional tests do not indicate the cause, the diagnosis is unexplained syncope. Of all patients with syncope patients in dedicated facilities, 18% to 20% had unexplained syncope.^2^, ^3^, ^4^ Our previous report also indicated that there was 23.9% incidence of all syncope.^5^ Insertable cardiac monitors (ICMs) allow for lengthy monitoring of cardiac rhythm and improved diagnostic yield among patients with unexplained syncope.^6^, ^7^, ^8^ Remote monitoring systems with ICMs have also become available. With ICMs, physicians can intensively monitor patients with unexplained syncope. In most cases, cardiac syncope is arrhythmic. Sick sinus syndrome, atrioventricular block, and paroxysmal supraventricular tachycardia (SVT) have been found in patients with unexplained syncope that is monitored by ICMs.^9^

When diagnosed through the use of ICMs, bradycardia, supraventricular tachycardia and ventricular tachycardia must be treated aggressively. In Western countries, patients who have unexplained syncope have been reported to have predictors for bradycardia that necessitates placement of a pacemaker^10^, ^11^, ^12^; this phenomenon has not been reported in Asian countries. In addition, the predictors for SVT have not been reported. If these predictors can be clarified, clinicians could provide more specific targeted monitoring. We therefore aimed to identify these predictors.

## 2 METHODS

We retrospectively reviewed medical records of consecutive patients who received ICMs to monitor unexplained syncope in three hospital facilities in Japan (Showa University Hospital, Showa University Fujigaoka Hospital, and Showa University Koto Toyosu Hospital) between January 1, 2009, and December 31, 2018. These patients underwent evaluation that consisted of documentation of a detailed history, physical examination, blood examination, and 12‐lead electrocardiography (ECG) at the time of consultation. These patients underwent the following tests if indicated: prolonged ECG (24 h to 7 days with Holter ECG recording or ECG monitoring during hospitalization), echocardiography, brain magnetic resonance imaging (MRI) or computed tomography (CT), head‐up tilt testing (include carotid sinus massage), echocardiography, treadmill stress testing, electrophysiological study, coronary angiography or electroencephalography (EEG). An ICM device (Reveal® DX, Reveal® XT, or Reveal® LINQ; Medtronic, Minneapolis, MN, USA) was placed subcutaneously in the left pectoral region in each patient in accordance with national guidelines^13^, ^14^ (2009 or 2018 guidelines of the European Society of Cardiology). The ICM was able to record three manual activations of 7.5 min and automatic activations that were programmed as the following: (1) rapid ventricular tachycardia (RR interval of <260 ms in at least 30 of 40 consecutive beats), (2) ventricular tachycardia (RR interval of 261 to 340 ms in 16 consecutive beats), (3) pause (>3.0 s) and (4) bradycardia (heart rate of <30/min in four consecutive beats).

ICM data were analyzed after each event, or, if no event occurred, patients were monitored routinely every 3 months. The data were downloaded from the ICMs and filed in the medical record. Some specialists in cardiac medicine used the ICM data to diagnose bradycardia and SVT.

We reviewed the rates of bradycardia and SVT among the patients with unexplained syncope monitored by ICM. We also reviewed the medical records of participants for clinical history, including activity at the time of syncope, situation and characteristics of syncope, comorbid conditions (e.g., hypertension, diabetes mellitus, dyslipidemia, prior stroke/transient ischemic attack, atrial fibrillation), etiology (e.g., congestive heart failure, ischemic heart disease, and other cardiomyopathy), medications, cardiac and neurological examination findings, blood pressure, body mass index, smoking history, and ECG results. Abnormal ECG findings were defined as sinus bradycardia (heart rate, <50 beats per minute [bpm]), PR interval of 200 ms or longer, left‐axis deviation, complete right bundle brunch block, premature ventricular contraction, Brugada‐type pattern, left ventricular hypertrophy pattern, long QT interval (>440 ms), and bifascicular block. We excluded data from patients who could not be monitored, such as those who changed hospitals during the 3 months of the study. Informed consent to participate in the study was obtained from each patient, and the study protocol was approved by the Ethics Committee of Showa University, Japan. The reference number is 3138.

### 2.1 Statistical analysis

Data are reported as means ± standard deviations. Continuous and categorical variables were compared by means of the Mann–Whitney *U* test or chi‐squared test, as appropriate. We performed Cox's stepwise logistic regression analysis to identify significant independent predictors that occurred during syncope and cardiovascular events and that were prognostic for bradycardia and SVT, and we calculated the odds ratios (ORs) and 95% confidence intervals (CIs) are presented. Using Kaplan–Meier curves, we analyzed the diagnosis on the day from ICM placed. We considered *p* values of less than .05 to be statistically significant. JMP software version 14.0 (SAS, Cary, NC, USA) was used for the analysis.

## 3 RESULTS

We reviewed the medical records of 140 consecutive patients, of whom six were excluded because they changed hospitals during the study period. Two more patients were excluded because their ICMs had to be removed prematurely as a result of infection or skin erosion. The remaining 132 patients were enrolled for further study and their clinical characteristics are shown in Table 1(A)–(C).

**TABLE 1:** Patient characteristics, situation of syncope and comparison of patients with and without Bradycardia and SVT

A							
	Total Patients	Bradycardia			SVT		
		Bradycardia (−)	Bradycardia (+)	*p*	SVT (−)	SVT (+)	*p*
*N*	132	113	19		124	8	
Baseline characteristics							
Age > 75 years	53 (40%)	45 (40%)	8 (42%)	N.S.	48 (39%)	5 (63%)	N.S.
Men	90 (68%)	74 (65%)	16 (84%)	N.S.	86 (69%)	4 (50%)	N.S.
First syncope	40 (30%)	35 (31%)	5 (26%)	N.S.	37 (30%)	3 (38%)	N.S.
Injury	41 (31%)	35 (31%)	6 (32%)	N.S.	39 (31%)	2 (25%)	N.S.
Hypertension	67 (51%)	54 (48%)	13 (68%)	N.S.	63 (51%)	4 (50%)	N.S.
Diabetes mellitus	25 (19%)	20 (18%)	5 (26%)	N.S.	24 (19%)	1 (13%)	N.S.
Malignancy	21 (16%)	17 (15%)	4 (21%)	N.S.	20 (16%)	1 (13%)	N.S.
Smoking	50 (38%)	41 (36%)	9 (47%)	N.S.	48 (39%)	2 (25%)	N.S.
BMI > 25	23 (17%)	17 (15%)	6 (32%)	N.S.	22 (18%)	1 (13%)	N.S.
SBP < 100 mmHg	5 (4%)	5 (4%)	0 (0%)	N.S.	5 (4%)	0 (0%)	N.S.
History of stroke	14 (11%)	13 (12%)	1 (5%)	N.S.	14 (11%)	0 (0%)	N.S.
History of af	25 (19%)	21 (19%)	4 (21%)	N.S.	20 (16%)	5 (63%)	<.05
Ejection function<50%	9 (7%)	9 (8%)	0 (0%)	N.S.	9 (7%)	0 (0%)	N.S.
Coronary disease	20 (15%)	16 (14%)	4 (21%)	N.S.	20 (16%)	0 (0%)	N.S.
Situation of syncope							
During effort	48 (36%)	37 (33%)	11 (58%)	<.05	46 (37%)	2 (25%)	N.S.
While supine	6 (5%)	6 (5%)	0 (0%)	N.S.	6 (5%)	0 (0%)	N.S.
Urination or defecation	13 (10%)	12 (11%)	1 (5%)	N.S.	12 (10%)	1 (13%)	N.S.
Drinking	9 (7%)	9 (8%)	0 (0%)	N.S.	9 (7%)	0 (0%)	N.S.
While taking a bath	9 (7%)	9 (8%)	0 (0%)	N.S.	9 (7%)	0 (0%)	N.S.
While driving	5 (4%)	5 (4%)	0 (0%)	N.S.	5 (4%)	0 (0%)	N.S.

B: Patient characteristics, characteristics of syncope, abnormal ECG and comparison of patients with and without Bradycardia and SVT							
	Total patients	Bradycardia			SVT		
		Bradycardia (−)	Bradycardia (+)	*p*	SVT (−)	SVT (+)	*p*
*N*	132	113	19		124	8	
Characteristics of syncope							
Prodromal symptoms	66 (50%)	56 (50%)	10 (53%)	N.S.	62 (50%)	4 (50%)	N.S.
Palpitation/chest uncomfortable	17 (13%)	14 (12%)	3 (16%)	N.S.	13 (10%)	4 (50%)	<.05
Vertigo	8 (6%)	7 (6%)	1 (5%)	N.S.	8 (6%)	0 (0%)	N.S.
Asthenia	4 (3%)	4 (4%)	0 (0%)	N.S.	3 (2%)	1 (13%)	N.S.
Diaphoresis	7 (5%)	6 (5%)	1 (5%)	N.S.	6 (5%)	1 (13%)	N.S.
Blurred vision	9 (7%)	8 (7%)	1 (5%)	N.S.	9 (7%)	0 (0%)	N.S.
Nausea	14 (11%)	13 (12%)	1 (5%)	N.S.	14 (11%)	0 (0%)	N.S.
Dyspnea	3 (2%)	3 (3%)	0 (0%)	N.S.	2 (2%)	1 (13%)	<.05
Stagger	6 (5%)	6 (5%)	0 (0%)	N.S.	6 (5%)	0 (0%)	N.S.
Convulsion	6 (5%)	6 (5%)	0 (0%)	N.S.	6 (5%)	0 (0%)	N.S.
Abnormal ECG	75 (58%)	64 (57%)	11 (58%)	N.S.	69 (56%)	6 (75%)	N.S.
Sinus bradycardia (HR < 50/min)	7 (5%)	5 (4%)	2 (11%)	N.S.	6 (5%)	1 (13%)	N.S.
PR≥200 ms	12 (9%)	10 (9%)	2 (11%)	N.S.	11 (9%)	1 (13%)	N.S.
Left axis deviation	11 (8%)	11 (10%)	0 (0%)	N.S.	10 (8%)	1 (13%)	N.S.
CRBBB	13 (10%)	10 (9%)	3 (16%)	N.S.	12 (10%)	1 (13%)	N.S.
PVC	3 (2%)	3 (3%)	0 (0%)	N.S.	3 (2%)	0 (0%)	N.S.
Brugada type pattern	6 (5%)	6 (5%)	0 (0%)	N.S.	6 (5%)	0 (0%)	N.S.
Long QT	4 (3%)	3 (3%)	1 (5%)	N.S.	4 (3%)	0 (0%)	N.S.
LVH pattern	5 (4%)	5 (4%)	0 (0%)	N.S.	5 (4%)	0 (0%)	N.S.
Bifascicular block	9 (7%)	6 (5%)	3 (16%)	N.S.	9 (7%)	0 (0%)	N.S.

C: Patient characteristics, medicine and comparison of patients with and without Bradycardia and SVT							
	Total patients	Bradycardia			SVT		
		Bradycardia (−)	Bradycardia (+)	*p*	SVT (−)	SVT (+)	*p*
N	132	113	19		124	8	
Medication							
Beta blocker	23 (17%)	19 (17%)	4 (21%)	N.S.	20 (16%)	3 (38%)	N.S.
Alpha blocker	8 (6%)	6 (5%)	2 (11%)	N.S.	8 (6%)	0 (0%)	N.S.
ACEI/ARB	39 (30%)	31 (27%)	8 (42%)	N.S.	37 (30%)	2 (25%)	N.S.
Diuretic	14 (11%)	13 (12%)	1 (5%)	N.S.	13 (10%)	1 (13%)	N.S.
Calcium channel blocker	42 (32%)	34 (30%)	8 (42%)	N.S.	39 (31%)	3 (38%)	N.S.
Coronary vasodilator	14 (11%)	13 (12%)	1 (5%)	N.S.	14 (11%)	0 (0%)	N.S.
Anti‐depressant	14 (11%)	13 (12%)	1 (5%)	N.S.	12 (10%)	2 (25%)	N.S.
Statin	38 (29%)	30 (27%)	8 (42%)	N.S.	37 (30%)	1 (13%)	N.S.

*Note*: Electrocardiogram (ECG) was considered abnormal if there were rhythm abnormalities, sinus bradycardia (heart rate, <50 beats per minute [bpm]), PR interval of 200 ms or longer, left‐axis deviation, complete right bundle brunch block, premature ventricular contraction, Brugada‐type pattern, left ventricular hypertrophy pattern, long QT interval (>440 ms), and bifascicular block.

Abbreviations: ACEI, angiotensin‐converting enzyme inhibitor; af, atrial fibrillation; ARB, angiotensin receptor blocker; BMI, body mass index; CRBBB, complete right bundle brunch block; PVC, premature ventricular contraction; ECG, electrocardiography; sBP, systolic blood pressure; SVT, supraventricular tachycardia.

The mean age of participants was 65 ± 20 years (range, 9 to 92 years), and 53 (40%) were 75 years of age or older. Ninety patients (68%) were men. Comorbid conditions included hypertension in 67 patients (51%), diabetes mellitus in 25 (19%), history of atrial fibrillation in 25 (19%) and coronary artery disease in 20 (15%). Forty‐one patients (31%) suffered major or minor injury during their syncopal events. During episodes of syncope, 48 patients (36%) were engaging in effortful activity, and 6 patients (5%) were supine. Prodromal symptoms were present in 66 patients (50%). The symptoms included palpitation or chest discomfort, in 17 patients (13%), vertigo in 8 (6%), and blurred vision in 9 (7%; Table 1(A),(B)).

**FIGURE 1:**
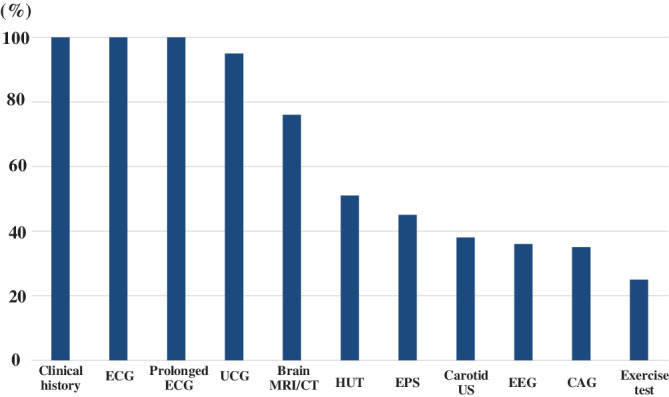
Workup before placement of insertable cardiac monitor. CAG, coronary angiography; CT, computed tomography; ECG, electrocardiography; EEG, electroencephalography; EPS, electrophysiological study; HUT, head‐up tilt test; MRI, magnetic resonance imaging; UCG, ultrasound cardiography; US, ultrasound

Figure 1 shows examinations for syncope diagnosis before ICM placement. Clinical history, 12‐lead ECG, and prolonged ECG were performed in all patients. Ultrasound cardiography was performed in 126 patients (95%); MRI/CT, in 101 (77%); head‐up tilt testing, in 67 (51%); electrophysiology study, in 58 (44%); carotid ultrasonography, in 50 (38%); EEG study, in 48 (36%); coronary angiography, in 45 (34%); and exercise stress testing, in 33 (25%).

**FIGURE 2:**
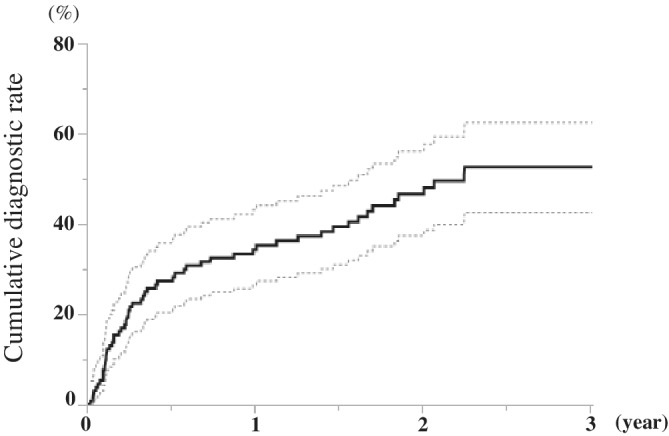
Cumulative diagnostic rate in the total patients. The dashed line reflects the 95% confidence interval

The median length of follow‐up after ICM placement was 17 months. No patients with unexplained syncope died during the observation period. Of the 132 patients who received an ICM, 19 (14%) received a diagnosis of bradycardia; 8 (6%), SVT (mean heart rate, 194 ± 28 bpm); 2 (2%), ventricular tachycardia; and 28 (21/%), noncardiac syncope (Table 2). Among the 132 patients, the total estimated diagnostic rates were 34% and 48% at 1 and 2 years, respectively (Figure 2).

**TABLE 2:** Diagnosed syncope during observation period

Diagnosed syncope	*N*
Cardiac syncope	
Bradycardia	19 (14%)
Supraventricular tachycardia (SVT)	8 (6%)
Ventricular tachycardia	2 (2%)
Non‐cardiac syncope	28 (21%)
Total	57 (42%)

**FIGURE 3:**
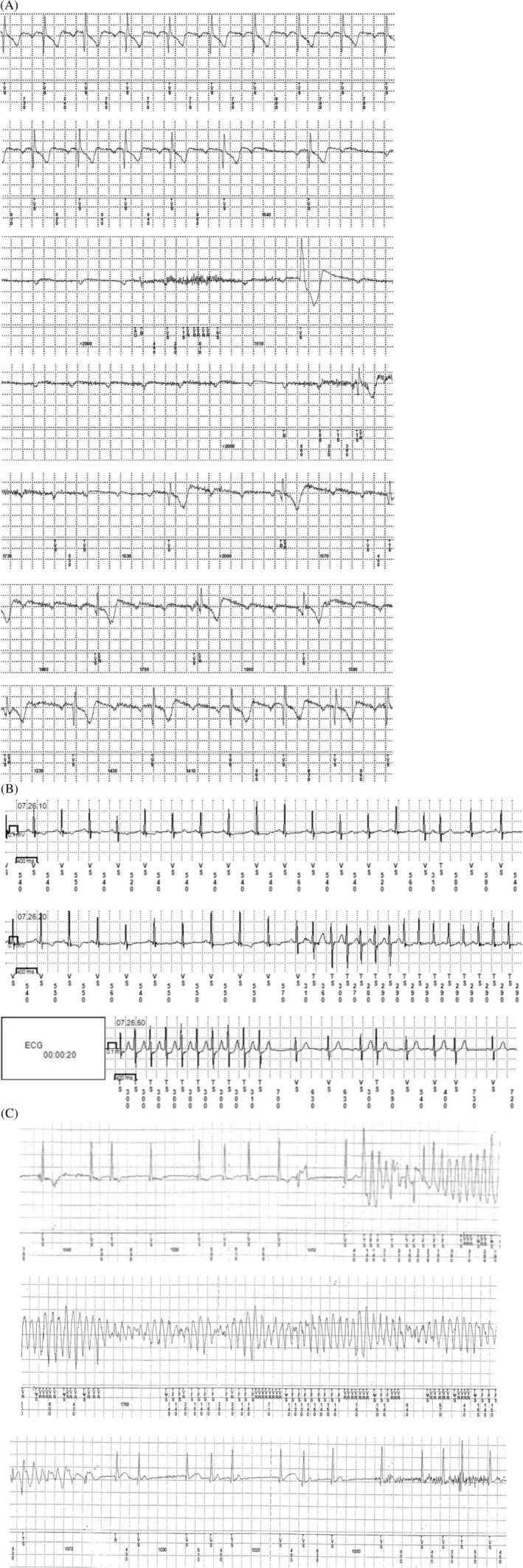
(A) Downloaded rhythm strip from 73 years old patient with two previous episodes of syncope with injury. One month after implantation of insertable cardiac monitor (ICM), patient had recurrent syncope during walking, and ECG captured paroxysmal atrioventricular block. (B) Seventy‐years‐old, female. She had two previous episodes of syncope with prodrome of palpitation during washing and cooking. Three months after implantation of ICM, ICM documented supraventricular tachycardia (SVT) during pre‐syncope with palpitation. Atrial flutter was induced by electrophysiology test, and we performed catheter ablation. (C) Eleven‐years‐old, female. She developed recurrence of syncope once a year from 5 years old during playing or tests at school. One and a‐half years after implantation of ICM, she had syncope while riding a roller coaster at the amusement park. Downloaded electrocardiography (ECG) showed polymorphic VT starting from short‐long‐short pattern, and QT interval change in sinus rhythm after spontaneously termination. After admission, concealed long QT syndrome was diagnosed by catecholamine stress test

Of the 19 patients with bradycardia, 10 patients had sick sinus syndrome and 9 had atrioventricular block; all 19 of these patients received pacemakers. Documented typical bradycardia ECG findings are shown in Figure 3(A). Of the 8 patients with SVT, 3 had atrial flutter, 4 had atrial tachycardia (including atrioventricular node reentry tachycardia and atrioventricular reentry tachycardia), and 1 had paroxysmal atrial fibrillation; all 8 underwent catheter ablation therapy. Documented typical SVT ECG findings are shown in Figure 3(B). Because the ventricular tachycardia was lethal arrhythmia, the 2 patients with ventricular tachycardia received implantable cardiac defibrillators. These arrhythmias had induced syncope and, fortunately, had terminated spontaneously. Ventricular tachycardia took the form of concealed long QT in 1 patient (Figure 3(C)) and was caused by idiopathic ventricular fibrillation in 1 patient.

Syncope during effort (in 58%; *p* < .05) was significantly more frequent in patients with bradycardia who needed a pacemaker (Table 1(A)) Stepwise logistic regression analysis indicated that syncope during effort (OR = 3.41; 95% CI, 1.21 to 9.6; *p* = .02) was an independent predictor for bradycardia that necessitated a pacemaker (Table 3). Patients with SVT who needed catheter ablation, in comparison with patients without SVT, more frequently experienced palpitation before syncope (50% versus 10%; *p* < .05) and had a history of atrial fibrillation (63% vs. 16%; *p* < .05) (Table 1(A),(B)). Palpitation before syncope (OR = 9.46; 95% CI, 1.78 to 50.10; *p* = .008) and history of atrial fibrillation (OR = 10.1; 95% CI, 1.96 to 52.45; *p* = .006) were identified as significant independent predictors for SVT (Table 3).

**TABLE 3:** Independent clinical predictors of bradycardia and supraventricular tachycardia necessitating therapy

Bradycardia Independent predictors of bradycardia	19 (14%) syncope during effort (OR 3.41; 95%CI 1.21–9.6, *p* = .02)
Supraventricular tachycardia Independent predictors of supraventricular tachycardia	8 (6%) Palpitation before syncope (OR 9.46; 95%CI 1.78–50.10, *p* = .008) History of atrial fibrillation (OR 10.1; 95%CI 1.96–52.45, *p* = .006)

Abbreviations: OR, odds ratio; CI, confidence interval.

## 4 DISCUSSION

We showed that syncope during effort, and palpitation, atrial fibrillation were independent predictors for bradycardia and for SVT. The main causes of syncope are known to be bradycardia, tachycardia (including SVT and ventricular tachycardia), noncardiac causes (including reflex syncope and orthostatic hypotension).^9^, ^15^ The arrhythmias must be aggressively treated because they affect quality of life and increase the risk of mortality.

ICMs are useful devices for diagnosing unexplained syncope. The annual cumulative diagnostic rate was calculated to be 43% to 50% over a maximum follow‐up period of 2 years in previous reports in Western populations.^6^, ^7^, ^8^ In those reports, the diagnostic rate increased rapidly during the 6 months period after ICM placement and linearly during the subsequent period.

Predictors of bradycardia have been previously reported in Western nations^10^, ^11^ but not in Japan. In a previous study, Ahmed et al reported that independent predictors for bradycardia that necessitated pacemaker implantation were female gender, age of more than 75 years, PR interval longer than 200 ms, and injury during syncope.^11^ In another study, Palmisano et al reported that age of more than 75 years, injury during syncope, and bradycardia on ECG were independent predictors for bradycardia that necessitated pacemaker implantation.^10^ Moreover, in both studies, the investigators reported that the presence of multiple predictors significantly increased the possibility that affected patients would need pacemaker implantation. Those results, however, differed from ours. Some reasons are that Japanese and Western clinical settings may be different; the definition unexplained syncope may differ; and there may be physiological differences among various races. In addition, fewer Japanese patients with unexplained syncope may agree to have ICMs placed.^5^ However, because syncope during effort has been reported to be a predictor for suspected cardiac syncope,^16^ we believe that our finding of syncope as a predictor of bradycardias necessitating pacemaker implantation is related.

To the best of our knowledge, no previous reports have elucidated the predictors of SVT in patients with unexplained syncope monitored by ICM. SVT can lead to reduced cardiac output and syncope because of increased ventricular rate. In most cases of SVT, however, the heart rate is not rapid enough to impair ventricular function and cardiac output.^17^ In a previous study, 20% of patients with SVT had at least episode of syncope which is preceded by palpitations. Multivariate analysis showed that heart rate ≥ 170 beats/min was the only independent predictor for syncope.^18^ The mean rate of SVT induced syncope was 194 bpm, most of patients was above 170 bpm in present study. The reason is considered that none of registered patients have severe structural heart disease such as low ejection fraction and cardiomyopathy. Syncope symptom is generally rare in patients with SVT; symptoms mostly reflect palpitation.^18^ We also found a strong connection between SVT and palpitation in patients with unexplained syncope monitored by ICM in our study.

A history of atrial fibrillation was significantly more common among patients with SVT in our study. Atrial fibrillation is known to coexist with paroxysmal supraventricular tachycardia (i.e., atrioventricular node reentry tachycardia, atrioventricular reentry tachycardia, and atrial tachycardia) and atrial flutter.^19^, ^20^, ^21^ To induce atrial fibrillation after ablation in electrophysiological test, an atrial burst pacing method is needed.^22^ A rapid atrial response in SVT‐induced syncope may induce atrial fibrillation. Additionally, atrial fibrillation causes electrical, contractile, and structural remodeling of the left atrium, and this remodeling is known to lead to mechanical dysfunction and enlargement of the left atrium.^23^ The dysfunction and enlargement of left atrium may accelerate hemodynamic instability by impairing ventricular function and cardiac output during episodes of SVT. A history of atrial fibrillation may be a significant predictor for SVT in cases of unexplained syncope.

Meanwhile, in bradycardia‐tachycardia syndrome representative of severe sick sinus syndrome, most episodes of tachycardia are caused by atrial fibrillation. However, a history of atrial fibrillation was not a significant predictor for bradycardia that necessitated pacemaker implantation. A history of atrial fibrillation was a predictor more for syncope with SVT than bradycardia.

### 4.1 Safety

Of the 132 patients in unexplained syncope monitored by ICM, two patients (2%) experienced ventricular tachycardia. All episodes of ventricular tachycardia were potentially lethal arrhythmias that induced syncope and spontaneously terminated. Peter et al. reported that of 173 patients with unexplained syncope monitored by ICM, two patients received implantable cardiac defibrillators because of ventricular tachycardia.^12^ In an earlier report, 0% to 13% of patients with unexplained syncope monitored by ICM had ventricular tachycardia.^10^, ^24^, ^25^ Although no patients died during observation period in our study, a minority of patients with unexplained syncope monitored by ICM did die in previous studies.^11^, ^12^ Clinicians should remember that patients with unexplained syncope include those with potentially lethal ventricular tachycardia or other risks for mortality.

## 5 STUDY LIMITATIONS

This study was observational and retrospective, and the findings need to be confirmed in a larger and longer trial. In addition, our data appear to have a selection bias. This is dependent on physicians who diagnose unexplained syncope and on interpretation of ICM data. Indeed, we may have overidentified predictors because a few patients had bradycardia or SVT that necessitated therapy.

## 6 CONCLUSIONS

ICMs are useful devices for diagnosing unexplained syncope. Syncope during effort, and palpitations or history of atrial fibrillation were independent predictors for bradycardia and for SVT. We should carefully follow up of patients with these predictors.

## CONFLICT OF INTEREST

These all authors have no sources of funding or conflicts of interest to disclose.

## Data Availability

The data that support the findings of this study are available from the corresponding author upon reasonable request.
